# Collimator design for a clinical brain SPECT/MRI insert

**DOI:** 10.1186/2197-7364-1-S1-A21

**Published:** 2014-07-29

**Authors:** Debora Salvado, Kjell Erlandsson, Alexandre Bousse, Michele Occhipinti, Paolo Busca, Carlo Fiorini, Brian Hutton

**Affiliations:** The Institute of Nuclear Medicine, University College London, London, UK; Politecnico di Milano, Milano, Italy

This project's goal is to design a SPECT insert for a clinical MRI system for simultaneous brain SPECT/MR imaging. We assume the stationary SPECT insert will consist of two rings of ∼5x5-cm SiPM-based detectors insensitive to magnetic fields, with 0.8-mm intrinsic resolution. The maximum diameter is 44.5 cm, the minimum diameter is 33 cm to accommodate the patient and MRI receive/transmit coil, and the FOV has a 20 cm diameter.

We have compared eight collimator designs: single-, 2x2-, 3x3- and 5+2½- pinhole, and single-, 2-, 3- and 1+2½-slit slit-slat, where ½-pinholes/slits are shared between two detectors. Analytical geometric efficiency was calculated for an activity distribution corresponding to a human brain and a target resolution of 10 mm FWHM at the centre of the FOV. Noise-free data were simulated with and without depth-of-interaction (DOI) information, and reconstructed for uniform, Defrise, Derenzo, and Zubal brain phantoms. For DOI it is assumed that the crystal's first and second half can be differentiated.

Comparing the multi-pinhole and multi-slit slit-slat collimators, the former gives better reconstructed uniformity and trans-axial resolution, while the latter gives better axial resolution. Although the 2x2-pinhole and 2-slit designs give the highest sensitivities, they result in a sub-optimal utilization of the detector FOV. The best options are therefore the 5+2½-pinhole and the 1+2½-slit systems, with sensitivities of 4.9*10–4 and 4.0*10–4, respectively. The brain phantom reconstructions with multi-pinhole collimator are superior as compared to slit-slat, especially in terms of symmetry and realistic activity distribution. DOI information reduces artefacts and improves uniformity in geometric phantoms, although the difference is small for the brain phantom. These results favour a multi-pinhole configuration.

**Figure 1:**
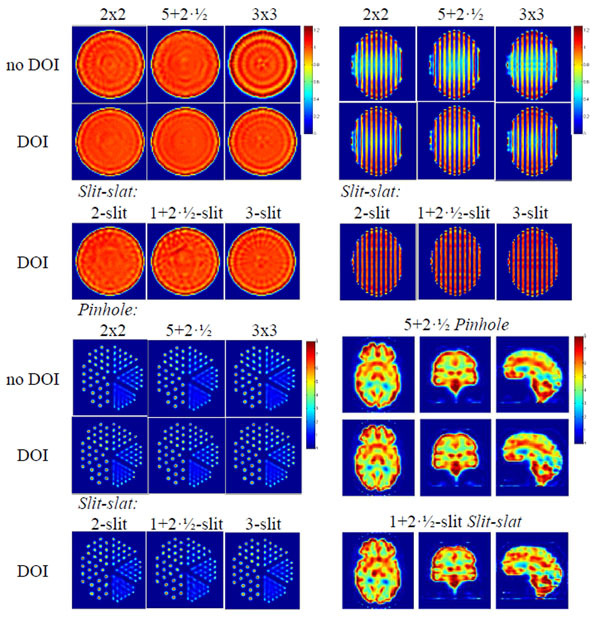
Top left: trans-axial sections of a reconstructed uniform phantom. Top right: sagittal sections of a reconstructed Defrise phantom. Bottom left: transaxial sections of a reconstructed Derenzo phantom. Bottom right: transaxial (left), coronal (middle) and sagittal (right column) sections of a reconstructed Zubal brain phantom.

